# Transradial Versus Transfemoral Approach for Coronary Angiography in Females with Prior Bypass Surgery

**DOI:** 10.7759/cureus.6797

**Published:** 2020-01-28

**Authors:** Ahmed Amro, Kanaan Mansoor, Mohammad Amro, Hisham Hirzallah, Amal Sobeih, Damir Kusmic, Ziad Abuhelwa, Majd Kanbour, Adee Elhamdani, Obadah Aqtash, Mehiar Elhamdani

**Affiliations:** 1 Cardiology, Marshall University, Huntington, USA; 2 Internal Medicine, Marshall University Joan C. Edwards School of Medicine, Huntington, USA; 3 Internal Medicine, Misr University for Science and Technology, Cairo, EGY; 4 Internal Medicine, Al-Najah University, Nablus, PSE; 5 Internal Medicine, Marshall University, Huntington, USA; 6 Internal Medicine, An-Najah National University, Nablus, PSE; 7 Internal Medicine, Allegheny Health Network, Pittsburgh, USA

**Keywords:** cabg, right radial artery access, femoral, contrast, fluoroscopy, radiation, female

## Abstract

Background

Multiple studies have shown that trans-radial access (TRA) for women undergoing coronary angiography/intervention (CA/I) has a lower risk of vascular access site complications as compared with trans-femoral access (TFA). In patients who had previously undergone coronary artery bypass grafting (CABG), studies also showed no significant difference between TRA and TFA in terms of contrast amount (CA), procedure time (PT), and fluoroscopy time (FT). However, those studies mainly included men. Limited information is available on the relative merits of TRA as compared with TFA for cardiac catheterization in females who previously undergone CABG. The purpose of this study was to determine the efficacy and safety of TRA versus TFA in women with prior CABG surgery who are undergoing CA/I in regard to CA, PT, and FT.

Methods

In this single-center retrospective cohort study, females with a history of CABG who underwent CA/I in the period from January 2013 to September 2016 were included. A total of 584 patients were included and divided into two groups: TRA group (49 patients) and TFA group (535 patients). The primary endpoints were CA, PT, and FT. The means for the primary outcomes were compared between the two using the independent t-score test.

Results

A total of 584 female patients with a history of CABG had cardiac catheterization from January 2013 to September 2016 at our center. Trans-femoral access accounted for 91.6% (n=535) of the patients while trans-radial access accounted for 8.4% (n=49) of the patients. A comparison of procedural variables between TRA and TFA revealed that there was no statistical significance in procedure time, fluoroscopy time, or the contrast volume. The access site crossover rate was 6.12% (n=3) from radial to femoral while there was a 0% rate in the femoral to radial access.

Conclusion

The key findings of this study suggest that in female patients with a prior history of CABG, TRA is an equally reliable and efficacious approach for both diagnostic angiography and intervention compared to TFA.

## Introduction

Cardiovascular disease (CVD) is the leading cause of mortality throughout the Western World for men and women alike [1-2]. Two-thirds of the women who suffer sudden cardiac death have no symptoms prior to their demise [3]. In 2006, the Women’s Ischemic Syndrome Evaluation (WISE) study highlighted the importance of heart disease in women [4]. Awareness of CVD has been on the rise but only 54% of women recognize CVD as the leading cause of death in 2012 [5]. Most of the guidelines, which dictate preventive strategies, diagnostic approaches, and management of CVD, rely on randomized clinical trials (RCT). Unfortunately, women represent only 30% of the subjects in the RCTs that are utilized as a basis for the American Heart Association (AHA) guidelines [1].

Cardiac catheterization is an important modality in assessing and treating coronary artery disease. In the past, trans-femoral access (TFA) was the benchmark for coronary intervention but, recently, the trans-radial access (TRA) has been shown to be as effective as TFA, if not more [6-7]. The radial vs femoral access for coronary intervention (RIVAL) trial reported that TRA had significantly fewer vascular complications, but there were no differences in the outcomes [8]. TRA is also favored because of its attributes of early recovery, ease of hemostasis, and early ambulation [9]. There have been multiple retrospective studies completed, which studied the efficacy of TRA in patients with a prior history of coronary artery bypass grafting (CABG); these studies reported that TRA is an equally feasible and efficient approach in comparison to TFA [9-10].

Most of the studies done in the past have been on the male population. One of the conclusive RADIAL-CABG trials on this topic did not have any female subjects [11]. Limited information is available on the relative merits of TRA as compared to TFA for cardiac catheterization in females who have previously undergone CABG. The purpose of this study was to determine the efficacy and safety of TRA versus TFA in women with prior CABG surgery who are undergoing coronary angiography/intervention in regard to contrast amount, procedure time, and fluoroscopy time.

## Materials and methods

Study design and patient population

Our study is a retrospective cohort conducted at a single center. The objective of the study was to compare the procedural variable of transradial and transfemoral cardiac catheterization in female patients who had previously undergone CABG surgery. Records from January 2013 to September 2016 were investigated. All female patients undergoing cardiac catheterization (both interventional and diagnostic) with a documented history of CABG were included in the study. There were no exclusion criteria. The study was approved by the institutional review board at Marshall University.

Procedure description

Transradial catheterization procedures utilized the right radial artery as the access site. After achieving access, intra-arterial nitroglycerin was administered via radial sheath to prevent arterial spasm and intravenous heparin was administered to prevent thrombosis. A post-procedure transradial band was placed at the access site to achieve hemostasis. Transfemoral catheterization procedures utilized either the right or left femoral artery. Post-procedure hemostasis was achieved by employing manual compression or a vascular closure device, at the behest of the operator. Access site preference was at the behest of the operator. This study utilized secondary data, and the exact reason for the specific access site was unavailable.

Endpoints

The primary endpoint of the study was the radiographic contrast volume administered during the procedure. Secondary endpoints were fluoroscopy time and the time required for the procedure (from time after the administration of the local anesthetic to the time required to complete the procedure with removal of the catheter).

Data collection

Patient and procedural information was extracted via a retrospective review of the patient’s electronic medical records.

Statistical analysis

Data were tabulated in SPSS version 20 (IBM Corp., Armonk, NY). Categorical variables, such as the sex, procedure type (diagnostic or therapeutic), and access site, were presented as percentages. Continuous variables, such as the volume of radiographic contrast administered during the procedure (primary endpoint), fluoroscopy time, and total procedure time, were presented as mean ± standard deviation (SD). The test of significance for continuous variables was the independent t-test. A P-value of < 0.05 was considered statistically significant.

## Results

A total of 584 female patients with a history of CABG had cardiac catheterization from January 2013 to September 2016 at our center. Trans-femoral access accounted for 91.6% (n=535) of the patients while trans-radial access accounted for 8.4% (n=49) of the patients. Baseline characteristics are enumerated in Table 1. The mean age of patients was 64.77 +/- 11.45 years. All of the patients were females. From the sample, 7.2% (n=42) of patients had ST-elevation myocardial infarction (STEMI), 19.7% (n=115) of patients had non-STEMI (NSTEMI), 51.9% (n=303) of patients had angina, and 20.2% (n=118) of patients had unstable angina, and 1% (n=6) of the patients had no indication stated in the chart for which there was a required cardiac catheterization. Patients that had diagnostic angiographic procedures composed 67.5% (n=394) of the sample while 32.6% (n= 190) of patients had percutaneous coronary interventions (PCI).

**Table 1:** Patient's characteristics BMI: body mass index; BSA: body surface area

Total N=584	Radial group n = 49	Femoral group n = 535	P-values
Age (years)	65.84	64.67	0.912
Height (cm)	162.18	160.45	
Weight (kg)	76.4	97.86	
BMI (kg/m^2^)	29.15	37.87	0.44
BSA (m^2^)	1.8	2.38	0.82
Number of grafts	2.71	2.75	0.62

Procedural outcomes are depicted in Table 2. With no statistical significance, compared with TFA, patients undergoing cardiac catheterization via TRA had a lower contrast use (119.39±58.87 vs 127.43±68.26, P=0.877), shorter procedure duration (39.63±26.43 min vs 42.30±26.87 min, P=0.258), and longer fluoroscopy time (17.13±22.63 min vs. 13.76±22.63 min, P=0.341). The access site crossover rate was 6.12% (n=3) from radial to femoral while there was a 0% rate in the femoral to radial access. All percutaneous coronary intervention (PCI) attempts were successful in both groups, and there were no major peri-procedural complications. There were no minor or major vascular complications in either group.

**Table 2:** Comparison of procedural variables between TRA and TFA TRA: trans-radial access; TFA: trans-femoral access

	Transradial access (n= 49)	Transfemoral access (n=535)	P-value
Contrast volume (ml)	119.39±58.87	127.43±68.26	0.258
Fluoroscopy time (min)	17.13±22.63	13.76±22.63	0.341
Procedural time (min)	39.63±26.43	42.30±26.87	0.887
Crossover	6.12% (n=3)	0% ( n=0)	0.292
Procedure type:			
*PCI	28.6% (n=14 )	32.9% (n=176)	
*Diagnostic	71.4% (n=35)	67.1% (n=359)	
Graft stenting	8	93	0.370
Native stenting	15	194	0.509
Stents	1.64±0.84	1.61±0.89	0.620
Access site Complications	0	0	

## Discussion

A well-known complication of cardiac catheterization is contrast-induced nephropathy (CIN). Furthermore, there is an increased risk of CIN with increased contrast amount and, therefore, less contrast amount means less incidence of CIN.

The primary endpoint of our study was to evaluate the contrast volume utilized during the catheterization procedure. The secondary endpoints were the total procedural time and fluoroscopy time. With respect to these procedural variables, our study revealed that there were no statistically significant differences in procedure time, fluoroscopy time, or the contrast volume, which conclude that both access sites are comparable and do not supersede one another. However, per our analysis, TRA does have a higher (6.12%) crossover rate in comparison to TFA (0%).

With the advent of cardiac catheterization in 1929, the femoral approach was the only access site available until 1989, when Campeau reported the first trans-radial diagnostic coronary catheterization [12-13]. Then, for the first time in 1993, Kiemeneij utilized TRA for percutaneous coronary intervention [14]. Subsequently, TRA gained further attention not only because it was a novel access site but also for its multiple positive attributes, such as lower rates of post-procedural complications, earlier hospital discharges, and better patient satisfaction [15-16]. In addition, procedural variables, such as contrast amounts, fluoroscopy time, and procedural duration, also needed consideration in comparison to TFA before TRA could be adapted for mainstream use. This study focused on the comparison of the latter mentioned variables, specifically in women with a prior history of CABG, a patient population that we feel has historically been neglected from such analysis.

Historically, studies on this topic have shown contradictory results both in relation to our study and each other. In 2013, the Radial-CABG Trial reported that a patient undergoing cardiac catheterization via TRA in comparison to TFA required more contrast volume (142 ± 39 ml vs. 171 ± 72 ml, p < 0.01), increased radiation exposure, and longer procedural time (21.9 ± 6.8 min vs. 34.2 ± 14.7 min, p < 0.01); this trial also reported a 17.2% crossover rate in the TRA arm of their study. Conversely, in 2016, Kedev et al. reported that TRA procedures required less contrast 100 (45-300) ml compared to TFA procedures 136 (46-350) ml (p-value 0.001) [7]. Rao et al. reported similar results, finding higher contrast volume use among TFA patients [17]. It is important to note, however, that the registry studies done by Kedev et al. and Rao et al. did not distinguish between CABG and non-CABG patients. For this reason, our study is best aligned for comparison with the RADIAL-CABG trial, although our particular focus is on the female population while RADIAL-CABG included only male patients.

Our study results contradict RADIAL-CABG in terms of the procedural variables, but we are in partial concordance due to the finding of a 6.12% (n=3) crossover rate in TRA to TFA [11]. In 2015, He et al. reported findings similar to our results and included an approximately 20% female population. He et al. highlighted that there was no difference between the procedure time and success rate and that the TRA was safe and feasible for patients with a prior history of CABG [9]. In 2016, a meta-analysis by Rigattieri et al. reported no statistical difference in procedural time (mean difference 3.24 minutes, 95% CI -1.76 to 8.25, p= 0.20), contrast volume (mean difference -2.58 ml, 95% CI -18.36 to 13.20, p =0.75), and fluoroscopy time (mean difference 0.62 minutes, 95% CI -0.83 to 2.07, p =0.40) when TFA is compared to TRA in patients with CABG; however, there was a higher crossover rate (OR 7.0, 95% CI 2.74 to 17.87, p <0.0001) in TRA procedures. Our analysis is in complete agreement with the aforementioned finding of Rigattieri et al [18].

Although TRA is gaining favor over the TFA, there are several caveats to consider. The most prevalent challenge when doing cardiac catheterization via the radial artery is obtaining access but once the access is achieved, the rate of successful cardiac catheterization is similar to TFA [15,19]. A study by Guédès et al. demonstrated that the majority of TRA procedure failures were due to the inability of the operator to puncture the artery; as per their recommendation, this hurdle can be surpassed if both radial arteries are attempted first prior to TFA conversion [20]. The caliber of the radial artery is small, as reported by Yoo et al. In men, the caliber is 2.69 ± 0.40 mm while it is 2.43 ± .038 mm in women. The small caliber of the radial artery makes it susceptible to spasm, which makes the manipulation of the catheter difficult and increases the risk of procedural failure or conversion [15,21-22]. Another factor that influences the TRA procedure success is lower catheter support, which decreases maneuverability and leads to procedure failure. Radial artery anatomical variation can play an integral role in the procedure outcome [15]. Valsecchi et al. studied the anatomical variation of the radial artery and its effect on the outcome of the procedure. The reported reasoning for failure and procedure crossover was due to a tortuous radial artery without stenosis, renal artery stenosis, hypoplastic radial artery, presence of radioulnar loop, the abnormal origin of the radial artery, and the retroesophageal origin of the subclavian artery [23].

Even though such challenges exist, TRA is still recommended, as per the guidelines of the AHA “Radial-First Strategy,” which should be considered when attempting cardiac catheterization [24]. There are multiple factors associated with TRA catheterization failure, namely, female sex, age (>75), history of CABG, cardiogenic shock, and short stature [24-27]. In light of this, the sample for our study was very high-risk, as we had included patients with all but one risk factor, specifically cardiogenic shock; even then, our results indicate that only 6.12% (n= 3) of the patients had to be converted to TFA from TRA while the remaining procedures were successful. Rathore et al. reported that a lower body mass index (BMI) was an independent factor for the failure of TRA but our analysis revealed that apart from the conversion, the remaining TRA procedures were successful although the BMI in our patients ranged from 20.14 to 50.99 with a mean of 29.15 ± 5.7 [28].

According to our data, 8.39% (n=49) patients had procedures via TRA while 91.6% (n= 535) patients had procedure via TFA. This disproportion in our data is due to the performing physician’s procedure preference. Upon further observation into the thought processes of various physicians, we were able to deduce that the access site of choice by most of the operators for CABG patients is trans-femoral but, even then, 8.39% of the patients had procedures via TRA. The success rate for the said TRA procedures was 100% with the exception of the cross-over patients. Furthermore, our study showed that all PCI attempts were successful in both groups, and there were no major peri-procedural complications. Also, there were no minor or major vascular complications in either group. However, given the fact that our study is a retrospective study, we suspect that there might be minor vascular complications that might have not been reported especially the majority of our patients were outpatients with same-day discharge.

The debate about using TRA in female patients is vital. It would be noteworthy to mention that the anatomical difference that makes TRA challenging in females also makes the TFA procedures prone to a higher risk of bleeding. In 2019, Kwok et al. reported higher crude bleeding rates in TFA (2.86%) and TRA (1.1%). This was attributed to the anatomy of the common femoral artery (CFA); the CFA is shorter and smaller in diameter, which inadvertently decreases the area for safe vascular puncture [29]. The Study of Access Site for Enhancement of PCI (SAFE-PCI) for women trial reported in 2018 that TRA and guided TFA presented no statistical differences in bleeding events and vascular complications [30]. In 2015, Pandie et al. reported the results of the RIVAL trial for women with ACS. According to their analysis, major vascular complications were significantly lower in women with TRA access: 3.1% vs 6.1% (hazard ratio (HR) 0.5; 95% CI 0.32 to 0.78 p=0.002) [6].

Gender bias in the current literature is a troublesome limitation [1]. Guidelines based on the current literature would be inadequate and subpar in guiding physicians to provide the most effective patient care. The most important resource for our topic is the meta-analysis by Rigattieri et al., which only has female representation in 21.17% of the sample [18]. Such discrepancies in sample collection hinder the generalizability of the findings. Although multiple patient studies have shown TRA to be as effective as TFA in regards to procedural outcomes and safer in regards to access site complications, TRA is not frequently employed for female patients with a prior history of CABG; this is evident in our study, as only 8.39% of the patients had procedures done via TRA.

Limitations

One of the major limitations of this study was the study design. This was a one-center, retrospective study with a lack of documentation provided about operator site preference. The disproportionate representation of the access site is also attributable to the retrospective nature of the study; this limited the statistical analysis of PCI in native and graft arteries, as the power of the sample was too low to achieve statistical significance. Authors have initially decided on not doing an analysis based on graft number, graft type, or any combinations of these because such groupings would lead to small sample sizes and would not be able to exclude other confounding factors. Finally, the secondary data were obtained from the catheterization laboratory; hence, the post-procedure complications were not evaluated.

## Conclusions

The key findings of this study suggest that in female patients with a prior history of CABG, TRA is an equally reliable and efficacious approach for both diagnostic angiography and intervention as compared to TFA. However, due to the limitations of this retrospective study, we recommend a multicenter randomized control trial to be done on this population of patients to compare TRA and TFA and include an analysis of the various CABG subgroups.
